# Comparative Study of SPA Mud from “Bacino Idrominerario Omogeneo dei Colli Euganei (B.I.O.C.E.)–Italy” and Industrially Optimized Mud for Skin Applications

**DOI:** 10.3390/life10060078

**Published:** 2020-05-26

**Authors:** Beatrice Bergamaschi, Laura Marzola, Matteo Radice, Stefano Manfredini, Erika Baldini, Chiara Beatrice Vicentini, Elena Marrocchino, Sonia Molesini, Paola Ziosi, Carmela Vaccaro, Silvia Vertuani

**Affiliations:** 1 Department of Life Science and Biotechnology, University of Ferrara, Master Course in Cosmetic Science and Tecnology, Via Fossato di Mortara 17–19, 44121 Ferrara, Italy; beatrice.bergamaschi@student.unife.it (B.B.); mv9@unife.it (L.M.); bldrke@unife.it (E.B.); vcc@unife.it (C.B.V.); vrs@unife.it (S.V.); 2 Department of Life Sciences, Universidad Estatal Amazónica, Km 2 ½ Puyo-Tena, 160101 Puyo, Equador; mradice@uea.edu.ec; 3 Department of Physics and Earth Sciences, University of Ferrara, Via Saragat 1, 44121 Ferrara, Italy; mrrlne@unife.it; 4 Ambrosialab s.r.l., via Mortara 171, 44121 Ferrara, Italy; sonia.molesini@ambrosialab.com (S.M.); paola.ziosi@ambrosialab.com (P.Z.)

**Keywords:** mud therapy, SPA muds, SPA treatments, mineralogy, trace elements, heavy metals, cosmetics, granulometry

## Abstract

The term “Salus per aquam (SPA) therapies” refers to therapeutic pathways that includes mud therapy. The therapeutic efficacy of a peloid depends on its chemical and mineralogical composition, as well as its technological properties. Considering the increasing use of clay-based products, it becomes essential to characterize peloids from a qualitative and quantitative point of view. Therefore, this research project aimed to develop a protocol that allows characterization of the chemical-physical composition of sludges collected from different areas of the Homogeneous Euganean Hills Hydromineral Basin (B.I.O.C.E.). The study established a comparative study both between different matrices and between the same matrices at different maturation times, including also a comparison with an industrialized product, that can be used at home, which maintains the characteristics of a natural mud. This research was developed studying the pH, grain size distribution, and chemical and mineralogical composition. Peloids are characterized by a neutral/basic pH and are divided into two categories from a granulometric point of view: The chemical composition allowed observation of numerous correlations between oxides present in the samples and to quantify the presence of heavy metals. Mineralogical analysis made it possible to identify and compare the composition of each sample, also according to the maturation time. Thanks to the methods adopted, important correlations were achieved.

## 1. Introduction

The use of clay minerals for therapeutic and cosmetic purposes has been documented since the beginning of civilization [1]. At the Estense court of Ferrara, the uses of SPA treatments are well documented and has attracted our attention, prompting us to start the present study, in the aim of revisiting the ancient traditional uses in light of the most recent techniques of investigation.

In particular, an incunabulum printed in Ferrara by the prototypographer André Belfort was examined. Michele Savonarola, a distinguished court doctor, grandfather of the most famous Girolamo, in “De balneis” refers to the whole Italian territory deals with each of the seven baths in the Euganean area with a precise and detailed analysis of their therapeutic properties [2,3].

The Euganean SPAs boasted a great prestige for their beneficial properties, so as to justify their use at court.

Sarah Bradford reports some court scenes: Alfonso was taking care of the waters, probably with the mud of Abano [4]. Lucrezia Borgia also used it in the garden next to the Castle, a complex equipped with various bathrooms, heating rooms, and a necessarium equipped with marble benches to sit on and marble stairs, from which you entered a bathroom stuffed with linen and heated by a stove. In addition to the water drawn from the ground beneath the city, mud and water barrels were brought from the SPA resorts around Padua, Abano, and San Bartolomeo for health care [5].

The term “SPA therapies” refers to therapeutic pathways that bring to the body the benefits of SPA water in composition with other treatments, including mud therapy, which consists of the application of SPA mud, i.e., mud consisting of a solid clay mineral component, characterized by a specific chemical and granulometric composition and a liquid component, consisting of a mineral water with particular characteristics of the temperature and chemical composition. The SPA muds used for this type of treatment are peloids, defined by some authors as “a mature mud or a mature mud dispersion, having therapeutic or cosmetic properties, composed of a complex mixture of natural fine-grained materials of geological and/or biological origin, mineral or sea water and organic compounds deriving from biological metabolic activity” [6]. Considering the definition of peloids as sludges that have undergone a maturation process, it is known that the maturation is a necessary process to optimize the therapeutic characteristics of the mud [7]. It consists in allowing the sludge to age in special tanks, for a period of time, not less than 60 days (up to two years), the natural virgin mud with mineral, thermo-mineral, sea, or lake water depending on the location of the SPA. The mud can undergo periodic stirring and the water can be stagnant, regularly renewed or continuously flowing inside the tanks. The maturation process therefore consists of a treatment of the clay, during which the material acquires the characteristics that make it mature and therapeutically active. The modifications connected to the maturation process are strictly specific phenomena for each single SPA mud: Veniale et al. [8] highlighted that the granulometric profile of the mud does not undergo appreciable modifications, but, in some cases, the clay fraction (particles < 2 μm) decreases due to the agglomeration of the particles. Minguzzi et al. [9] and Sanchez et al. [10] found no significant differences when comparing raw sludge, mature sludge, and spent sludge, taking 10 main chemical elements and 23 trace elements as references.

The peloids can be classified (Table 1) according to their mineral and chemical composition [6] or, alternatively, according to the parameters characterizing the natural sediments that form them, which are the origin and composition of the solid phase, relative content of inorganic and organic material, chemical nature and temperature of the mineral water, and the mixing/maturation process with or without agitation.

The term “thermal therapies” refers to therapeutic and rehabilitative paths for several diseases. The approach to thermal therapy can be both internal and external, where external application includes balneotherapy, anthro-therapy, and mud therapy. Mud therapy means a session consisting of several steps, which involves the application of muds on the body surface.

The efficacy of thermal therapies has been exploited since ancient times and studies have been recently undertaken to demonstrate its therapeutic effects. From a clinical point of view, thermal therapy is considered useful in the treatment of diseases of the musculoskeletal system, such as osteoarthritis, rheumatoid arthritis, and rheumatological extra-articular diseases [11,12,13]. In addition, the effects of mud therapy are clinically recognized also in the dermatological field, for the treatment of skin diseases (acne, seborrhea, psoriasis), lipodystrophies, and cellulite [14]. Finally, muds are largely used for cosmetic treatments, and their effects generally attributed to the presence of a large amount of trace elements, their high adsorbing and ion exchange capacity, and other claimed properties but without consistent scientific support. Therefore, in order to improve the knowledge concerning the thermal therapies and correctly address the claimed effects of each SPA mud, a classification study is needed.

The therapeutic efficacy of a peloid is generally attributed to its chemical and mineralogical composition, as well as its technical properties. Indeed, some of them, such as the particle size, decrease during the maturation process [10]. Another aspect to consider is the evaluation of potential risks for human health, since peloids may contain elements with toxicological relevance (i.e., As, Cd, Co, Cr, Hg, Ni, Pb, Sb, Se, Te, Tl) that could be released during application. Summa and Tateo [15] confirmed that sweat, produced during a normal pelotherapy session, can extract small traces of dangerous chemicals from the peloid.

The presence in the mud of living species, such as algae (mainly cyanobacteria and diatoms), bacteria, protozoa, and biomaterials derived from their catabolic and anabolic activities, could also play a very important role in giving the SPA mud the known beneficial and curative properties.

In the literature, the attention on SPA treatments is mainly focused on their beneficial effects proven by several clinical studies [11,12,13,14], while the chemical and mineralogical study of SPA matrices remains in the background. Considering the increasing use of clay-based products, it becomes essential to characterize peloids from a qualitative and quantitative point of view [16,17]. Starting from the concept that the traditional and consolidated use over the millennia must be confirmed on the base on scientific evidence, we considered that knowledge of the composition of geo-materials is the starting point to reduce possible health risk factors and to deepen other complementary aspects, such as the optimization and functionalization of the product according to its intended use. This whole research project, therefore, aims to develop a protocol that allows, through consolidated analytical methods, the chemical-physical characterization of sludge collected from different areas of the Homogeneous Euganean Hills Hydromineral Basin (B.I.O.C.E.), setting up a comparative study both between different matrices and between the same matrices at different maturation times. This work is necessary because a systematic approach has never been described in the literature and very often, different muds are characterized on the basis of the mineralogical composition used as claim ingredients, for the health properties boasted by the SPA site itself. In addition, we compared a natural mud with an industrialized product, formulated in the laboratory to develop a reconstituted mud, which can be used at home, thus mimicking the characteristics of a natural mud.

## 2. Materials and Methods

### 2.1. Sludge Sampling

**Figure 1:**
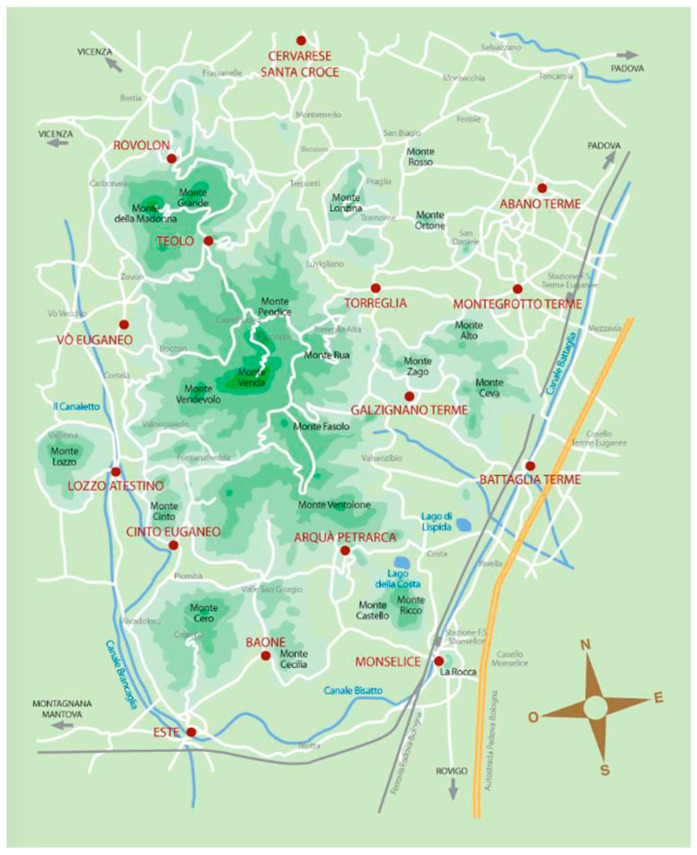
Euganean Hills Homogeneous Hydromineral Basin (B.I.O.C.E.) area.

The sampling of the natural SPA mud was carried out at the localities of Montegrotto, Galzignano, and Abano Terme, geographically located within the area of the Homogeneous Hydromineral Basin of the Euganean Hills (B.I.O.C.E.) (Figure 1). This area includes the territory of the municipalities of Abano Terme, Arquà Petrarca, Baone, Battaglia Terme, Due Carrare, Galzignano Terme, Monselice, Montegrotto Terme, Teolo, and Torreglia.

The first sampling of natural SPA mud was carried out at the “Grand Hotel Terme” in Montegrotto Terme and Galzignano Terme, and the second at the “Hotel President” located in Abano Terme. The samples of SPA mud were collected from tanks with different maturation times, in turn taken in three different points of the tank, respectively, for each on the right, center, and left, for a quantity of about 1 kg each. Before and after the analysis, the sludge samples were stored in the freezer.

The sludge collected at Galzignano terme is a mixture of sludge left to macerate in the decantation tanks added with sludge taken from continental environments with a geochemical imprint compatible with the sediments of the area. For this reason, the sludge is entirely composed of natural ingredients, but since it is a mixture from two different sources, it was defined as semi-synthetic for the purposes of nomenclature.

Subsequently, an “optimized” mud, composed of natural ingredients and water from the Euganean SPA basin, was formulated to obtain an industrializable product with chemical-physical properties similar to the natural SPA mud from the Euganean SPA basin but highly reproducible. The Euganean SPA basin was chosen as the target for its well-known curative properties [3]. The formulation of the optimized mud has the following INCI: “Aqua*, Solum Fullonum, Kaolin, Bentonite, Algae Extract, Magnesium Aluminum Silicate”.

A summary of the nomenclature adopted is reported in Table 2.

### 2.2. pH Determination

For each sample taken, pH measurement was carried out with a Crison-Micro pH 2000 pH-meter. Each measurement was repeated in triplicate.

### 2.3. Particle Size Analysis

The particle size analysis was performed using a sedimentation balance and an X-ray sedigraph (Micromeritics Sedigraph 5100) associated with the Sedimcol software, reprocessing the data according to Folk and Ward [18] notations.

A manual quartation was performed on all samples, using the opposite quarter method. The portions obtained were treated with 110 mL of H_2_O_2_ (16 volumes) to destroy any colloidal substances that could aggregate the particles. Subsequently, each sample was sifted wet (63-μm net sieve) to separate the sandy component from the silty-clay component. The sandy component was dried in an oven at 105 °C and further sieved (sieves with 2 mm and 63 μm opening), then divided into 5 equal parts with a quartator. The obtained aliquots were added to obtain a sample weighing between 2.7 and 3.2 g. The silty-clayey component was dried by sifting water using a siphon, then 30–35 g of sample was dried in an oven at 105 °C for at least 24 h. The difference in weight before and after drying reflected the amount of total water in the wet fraction.

To reduce any errors resulting from the experimental measurements, the actual density of the dried sludge was also determined using a helium pycnometer (Micromeritics Accupyc II 1340). This data was subsequently used as input to the sedigraph analysis.

### 2.4. Chemical Analysis by X-ray Fluorescence: XRF and Quantification of Heavy Metals

The chemical composition (both in major and in trace elements) of some pulverized, within an agate mill, mud samples was determined by X-ray fluorescence (XRF) using an ARL ADAVNT.XP spectrometer [19]. The samples were treated to remove the organic component by the dry way, so they were heated in the stove at 105 °C for 24 h (the determination of the sample weight before and after reflects the % water loss). Then, they were heated in muffle furnace at 500–550 °C for 24 h (the determination of the sample weight before and after reflects the % loss of organic matter). Finally, the samples were calcined in a muffle at 1000 °C for 24 h to remove the carbonate content.

With the powders deprived of the organic component, tablets were made on a boric acid support using a hydraulic press (calibration: *p* = 80 kg/mm^2^, t = 60 s). During compression, the powders were mixed with an organic binder, then evaporated after compression.

To confirm the results obtained with regard to the percentages of CaO, the analysis was deepened with a determination of loss on ignition (LOI), a parameter that expresses the loss in weight percentage of the clay and carbonates after calcination in a muffle at 1000 °C. This is a procedure used in laboratory practice to assess the organic matter and carbonate content of sediments [20,21]. Loss on ignition was measured by weighting before and after 12 h of calcination at 1050 °C.

From the chemical analysis, the amounts of heavy metals present in each sludge matrix were extrapolated and compared with reference values set by Health Canada, the European Pharmacopoeia, and the United States.

### 2.5. Mineralogical Characterization by X-ray Diffractometry (XRD)

X-ray diffraction allows the identification of crystalline compounds to trace both the nature of the substance and its crystalline form. Mineralogical characterization of samples powdered within an agate mill was carried out by X-ray diffraction (XRD) by means of a Philips PW1860/00 diffractometer (supplied by the Department of Earth Sciences of the University of Ferrara), using graphite-filtered CuKα radiation (1.54 Å) to identify the constituent mineralogical phases. Diffraction patterns were collected in the 2θ angular range 3–50°, with a 5 s/step (0.02 2θ). Then, the analysis was associated with the X-Powder software and the “Powder Diffraction File”, an electronic archive containing crystallographic information for more than 300,000 inorganic and organic phases. The processing of the acquired intensities and the correction of the matrix effect were performed according to the Lachance and Trail [22] model.

The results of all analyses performed are reported as a single sample for each sample tank. Preliminary investigations confirmed that there are no differences in the pH, particle size, and chemical and mineralogical composition between different points in the same tank.

## 3. Results and Discussion

### 3.1. pH Analysis

Table 3 shows the average pH value of the samples, obtained from the measurement in triplicate. The range is between 7.37 and 8.89, so the sludges are characterized by a neutral/lightly alkaline pH, due to the nature of the aluminum-silicates.

The pH does not change significantly as a function of the increasing maturation time (the pH values do not differ by more than 5%) or even between different hotel samples at the same maturation time (V2C, V2, both at 6 months of maturation). The “optimized” mud has more basic pH values than natural SPA muds, as does the semi-synthetic mixture (V4C), which shows higher pH values than the total natural SPA’s mud collected. Although the “optimized” mud has higher pH values than the natural ones, it is known in the literature that varying the quality of water in the muds has a significant influence on the final pH [8]. For this reason, further investigations of this aspect should be addressed to adjust the pH of the product to be more eudermic.

### 3.2. Particle Size Analysis

Loose sediments and sedimentary rocks consist of discrete particles and their characteristics and classifications are based on the texture and on chemical-mineralogical composition. The sediment, from a granulometric point of view, can be classified considering a size scale, among which the most adopted is the one proposed by Wentworth [23], according to which the clasts making up the sediment can be divided into six main classes.

The sediment is normally composed of one or more size classes and, for this reason, the size’s classification should take into account the relative abundances of each of the constituent classes. Shepard’s diagram [24] is used for classification purposes. Important properties are directly and indirectly linked to the grain size of the sediment: Air and water permeability, plasticity (and therefore workability), water retention capacity, and nutrient availability.

**Figure 2:**
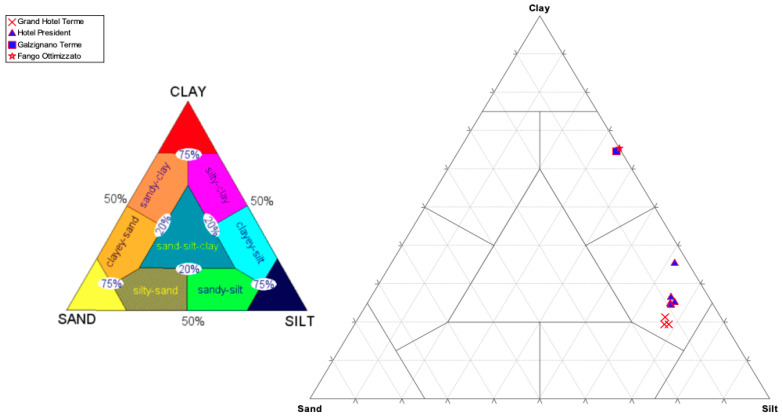
Particle size distribution represented according to Shepard’s diagram.

Figure 2 and Table 4 show respectively the distribution of the analyzed samples within Shepard’s triangle and their composition in percentage of sand, silt, and clay.

The grain size classes to which they belong are the following:

- “Clayey-silt”: With a prevalent silty component, represented on average by 65.42% of the total sample; followed by the values of clay with an average of 24.62% and those of sand (9.83%), which constitutes the minor component of the sample.
- “Silty-clay”: With a prevalent clay component, represented on average by 64.37% of the total sample; followed by silt values with an average of 34.41% and those of sand (1.24%), which is the minor component of the sample;

The samples taken from the Grand Hotel Terme and Hotel President are classified as clayey-silt, while the samples from Galzignano Terme and the optimized mud are classified as silty-clay.

The grain size analysis lends itself to several considerations, first of all the assessment of the skin feel. The ideal product should have the lowest possible sand content because, being the coarsest fraction, it could cause irritations or injuries to the skin. Moreover, a higher clay content is preferable compared to the silty component: The clay fraction determines, at the same volume, a greater specific surface area, giving the mud a greater reactivity (for example, a greater cation exchange capacity). Among all analyzed samples, from the granulometric point of view, the “optimized” sludge (FG) is the best one and can be elected as a reference product for the possible development of clay-based cosmetic products. It is classified as “silty-clay” and is composed of only 0.82% sand. In comparison with other studies reported in the literature [25], muds collected in the B.I.O.C.E. area have a finer particle size, so it is possible to affirm that these natural muds have a better quality and are gentler when applied on the skin.

The granulometric analysis permitted an understanding of whether the maturation process influences the granulometry of the mud itself. With regard to the SPA mud collected at the “Grand Hotel Terme” (VnC series), the particle size distribution is similar for all the samples; therefore, it can be said that the increase in maturation time from 3 weeks (V3C) to 1 month (V1C series) up to 6 months (V2C series) does not significantly influence the particle size of the mud.

For the SPA sludge collected at the “Hotel President” (Vn series), it is noted that, in the same way, the maturation process does not significantly influence the grain size. The samples of the V5 tank, despite being a “clayey-silt” like the others collected at the same SPA, contain a higher percentage of clay than the other tanks. Moreover, they have a significantly lower sand content, which is assumed to be due to the inevitable sedimentation process, which allows the deposit of sand at the bottom of the pool.

About the SPA mud collected at “Galzignano Terme” (V4C), since the clay fraction represents about 60% of the total sample, the classification of “silty-clay” given by Shepard’s triangle is confirmed.

Table 5 shows, instead, the granulometric distribution (wt%) of the sandy fraction. It is noted that the greatest contribution is given by fine sand (1.72%) and very fine sand (6.11%), which represent respectively 19.28% and 68.50% of the total sand component, while the components of very coarse sand, coarse sand, and medium sand represent respectively 1.01%, 4.26%, and 7.06% of the total sand component. It can be confirmed that the presence of the sandy fraction in natural SPA mud, following its application, does not cause irritative or abrasive problems since, according to the Udden–Wentworth classification (Table 6) [23], the sand dimensions corresponding to the classes “fine sand” and “very fine sand” are included in the grain size range 125–63 μm. Other studies on the grain size of natural SPA mud also confirm this theory [25].

To increase the functionality of the product, it is preferable to use, as already mentioned, clays and sludges composed mainly of clay. Depending on this, it is more preferable to use “optimized” mud, classified as “silty-clay”, than natural SPA mud samples, classified as “clayey-silt”.

### 3.3. Chemical Analysis by X-ray Fluorescence (XRF) and Quantification of Heavy Metals

In Table 7, the values relative to the main oxides and those present in traces are reported, which have the following composition ranges: SiO_2_ = 50.59–58.62%, TiO_2_ = 0.48–0.82%, Al_2_O_3_ = 8.82–20.27%, Fe_2_O_3_ = 3.02–6.85%, MnO = 0.15–0.23%, MgO = 3.16–7.07%, CaO = 6.61–25.71%, Na_2_O = 1.07–1.80%, K_2_O = 2.15–2.34%, P_2_O_5_ = 0.11–5.72%, and LOI = 4.85–19.68%.

The samples analyzed show a positive correlation between SiO_2_% and Al_2_O_3_%, due to the nature of the clay composition sludge with a bimodal distribution. The SiO_2_% content is mainly related to the abundance of quartz due to its lack of correlation with the oxides of the other elements, as confirmed by the XRD analysis.

The group of samples with low concentrations of aluminum and silicon (natural SPA mud) are characterized by the presence of aluminum-silicates, such as clayey minerals. The second group (semi-synthetic sludge-Series Vn and “optimized sludge” -Series FG) is characterized, instead, by a high concentration of aluminum, related to higher concentrations of silicon. This suggests the presence of aluminum in other mineralogical phases, such as aluminum oxides and hydroxides, with the possibility of skin assimilation of the element itself. Furthermore, the XRF analysis confirms the classification of sludge previously made on a granulometric basis. In fact, the clay fraction, indicated by the presence of the Al_2_O_3_ percentage, is higher for V4C and FG series samples.

The natural SPA mud samples have a negative correlation between the CaO and Al_2_O content. The relative low concentrations in SiO_2_ and Al_2_O_3_, compared to the other samples, are related to the abundant presence of bioclasts, expressed by CaO %, mainly contained in carbonates (CaCO_3_). The LOI content, expressed as % values, confirms the high concentration of CaO in natural SPA mud, because it is correlated to the initial presence of whips and bioclasts detected in the natural samples, which are instead visually absent in the other samples.

There is a negative correlation between the MgO and SiO_2_ content and a higher magnesium concentration in the natural SPA sludge group. It can be assumed that these samples are richer in chlorite, amphibole, and smectite.

The positive correlation between the Rb ppm and Al_2_O_3_ content indicates the presence of clay sediments probably rich in illite. The variation between K_2_O, usually characterized by a geochemical behavior similar to Rb ppm, and Al_2_O_3_ contents shows that the presence of potassium is comparable between high and low aluminum content samples (V4C-FG series and natural sludge series, respectively). Generally, potassium is hosted in aluminum-silicate minerals; therefore, it can be confirmed that SPA sludge is illitic. In the group of semi-synthetics and optimized sludge, the presence of potassium is probably due to the presence of hydroxides.

There is also a positive correlation of the Fe_2_O_3_ and SiO_2_ content among all the samples. The presence of iron is probably due to the presence of sulphides.

The correlation between the MgO content and transition elements, such as nickel and chromium (values reported in Table 8), allows division of the samples into two main populations: The “optimized” sludge and the V4C series samples have higher nickel and chromium contents than the natural SPA sludge group (V1C, V2C, V3C, V1, V2, V3, V4, V5 series). Generally, magnesium silicates contain nickel and chromium; instead, in this case, the negative correlation between the oxide and transition metals indicates that the enrichment of these elements is due to the presence of nickel and chromium sulphides, or to adsorption phenomena in the clay minerals.

Furthermore, the concentrations of the elements are directly proportional to the concentration of Al_2_O_3_ (positive correlation), which expresses the tendency of these elements to concentrate within the fine fraction of the sample.

Taking into account that SPA sludges may contain potentially hazardous elements [7], the assessment of the chemical composition of the sample must take into account the assessment of the grain size profile, especially for samples classified as “clay-silty”, as the clay fraction determines, for the same volume, a greater specific surface area, giving the sludge a greater reactivity.

Consequently, if, on the one hand, this favors the exchange of macronutrients and micronutrients that are probably essential for the body [26], on the other hand, clay particles can control the mobility and bioavailability of potentially toxic elements (As, Cd, Co, Cr, Hg, Ni, Pb, Sb, Se, Te, Tl). With regard to this latter aspect, it is important to highlight the problem related to the presence of heavy metals: These elements are naturally present in rocks, soil, and water and they can be introduced in the processing of many raw materials. The concentration of Ba of the analyzed samples could be linked to the adsorption capacity of Euganean Hills clays towards mobile elements [27]. According to Cappuyns 2018 [28], despite relatively high pseudo-total concentrations (224.37–445.35 ppm), as compared to commonly investigated trace elements (Cd, Zn, Pb, Cu, Ni), Ba showed low mobility. This indicates that only a very small proportion of Ba is readily available for leaching or uptake. Leaching tests and extractions, developed to evaluate heavy metal mobility in soils, require careful interpretation when Ba is considered and are likely to not always be suited for Ba because of unintended side effects, such as precipitation reactions.

The data of the XRF analysis also allow an evaluation of, among the heavy metals mentioned above, the concentrations of Pb, Ni, Cr, and Co. However, it is important to highlight that the values collected from this analysis confirm the results reported in other studies [29], even if there is always some variability.

**Figure 3:**
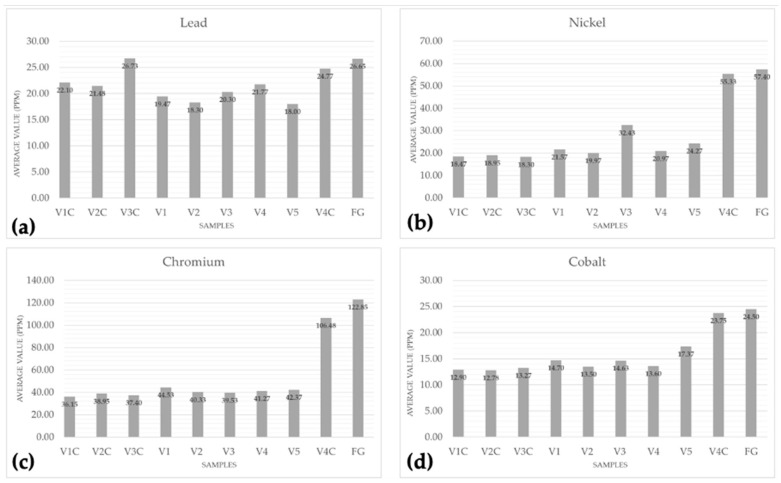
Histograms related to the heavy metals’ concentration in each sample. (**a**) Lead concentration (ppm): each sludge has a lead concentration higher than the limits set by Health Canada; at the same time, all samples remain within the limits set by the European and US Pharmacopoeia. (**b**) Nickel, (**c**) chromium, and (**d**) cobalt concentrations (ppm): all peloids have a cobalt concentration higher than the set references.

Since the current European Regulation (Annex II) [30], for the substances mentioned above, does not set either the maximum acceptable concentration or the toxicity threshold, we decided to use as a reference limit value for Pb the one set by the ICCR (International Cooperation on Cosmetics Regulation) [31] and Health Canada [32], which corresponds to 10 ppm. In addition, we took into account that, according to the European and US Pharmacopoeia, the amounts of Pb in bentonite should not exceed 40ppm [7]. The average values (ppm) relative to Pb of each sample were compared with these reference values in order to understand how much exceeds the concentration of the metal. In Figure 3a, it can be observed that no sample falls within the set limit, exceeding it by 1.5 to 2.7 times. On the other hand, according to the standards of the Pharmacopoeia, the levels of Pb remain within the acceptable limits, since they are all lower than 40ppm (it must be taken into account, however, that the limits imposed by the Pharmacopoeia refer to bentonites, which are clays, not peloids, but at the same time, peloids are partly composed of clays). These latter considerations for semi-synthetic and optimized sludge are somewhat in contrast with the fact that they are considered by consumers as being better than all natural ones because many are purified.

Particular attention was also paid to nickel, as it is considered the main cause of allergic contact dermatitis (CD). However, chromium and cobalt are also highly allergenic elements.

Although there is a lack of official limits on the concentration of heavy metals in cosmetics, the reference tolerance limit is conventionally set to 1 ppm. This value has been extrapolated on the basis of several studies involving subjects with CD, which have shown that most nickel-allergic patients do not show reactivity for concentrations below 1 ppm [33,34]. The mean values (ppm) for nickel, chromium, and cobalt of each sample were correlated with the reference value (Figure 3b–d): All samples, for all elements, were well above the threshold value of 1 ppm. In addition, it can be observed from the histograms that, for the three elements taken into consideration, the samples with the highest concentrations are the semi-synthetic sludge (V4C) and the optimized sludge (FG). As reported above, this an important issue that should be taken into account.

On the other hand, it is reasonable to think that the concentration of heavy metals varies according to the extraction basin. Indeed, in studies on muds extracted from other basins, the concentration for each metal is largely variable, ranging from similar to totally different values [25].

### 3.4. Mineralogical Characterization by X-ray Diffractometry (XRD)

X-ray diffractometry allows a qualitative and semi-quantitative evaluation of the different mineralogical phases present in the sample. This analytical technique is based on the properties of each crystal substance (reticular plans) to diffract the X-ray products by Coolidge vacuum tubes [35].

The mineralogical analysis allows the identification and comparison of the composition of each sample and the influence of the maturation process on the mineralogical composition.

Most samples show a simple mineralogical association, with a few exceptions. Quartz, calcite, and feldspar are always present, and dolomite is common, as well as phyllosilicates (chlorite and illite). In the “optimized” mud samples, kaolinite is also present.

**Figure 4:**
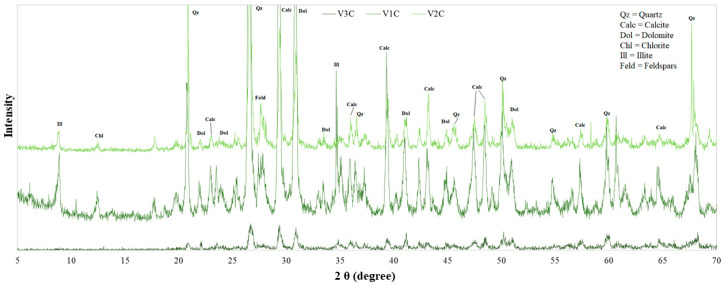
Comparison of the X-ray diffraction patterns of muds collected at the “Grand Hotel Terme” spa resort.

**Figure 5:**
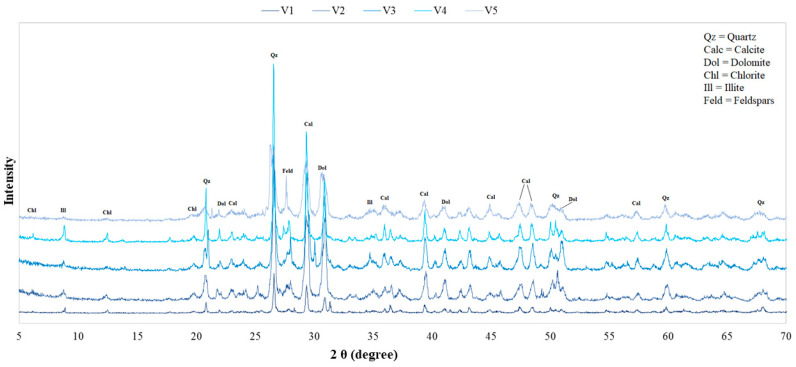
Comparison of the XRD patterns of muds collected at the “Hotel President” SPA resort.

To understand if the maturation process influences the mineralogy of the SPA mud, the X-ray diffraction patterns of the samples were superimposed in correspondence with the different maturation times: Figure 4 and Figure 5 show, respectively, the mineralogical compositions of the “Grand Hotel Terme” and “Hotel President” samples. The overall maturation process does not imply a significant change in the mineralogical composition, as also confirmed by other studies carried out in different SPAs [15,36].

**Figure 6:**
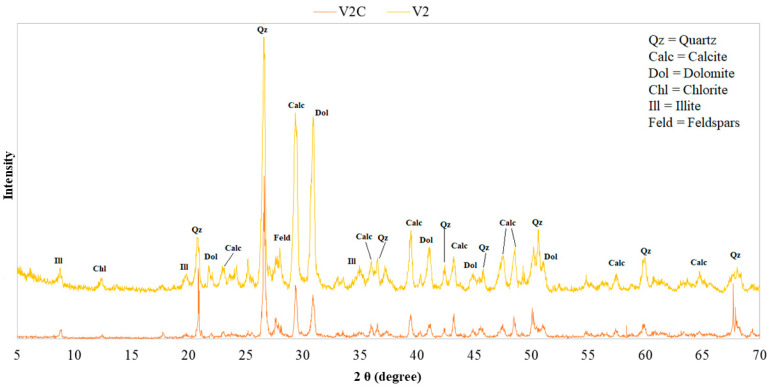
Comparison of the XRD patterns of SPA muds at the same time of maturation, collected at two different spas (“Grand Hotel Terme” and “Hotel President”).

The mineralogical composition was also compared between sludge at the same time of maturation (V2C, V2), taken from two different hotels (“Grand Hotel Terme” and “Hotel President”), which have two separate wells. As shown in Figure 6, there is no significant change in composition, confirming an expected fact: The geological characteristics of the territory do not change in such a circumscribed area as the Euganean SPA basin.

**Figure 7:**
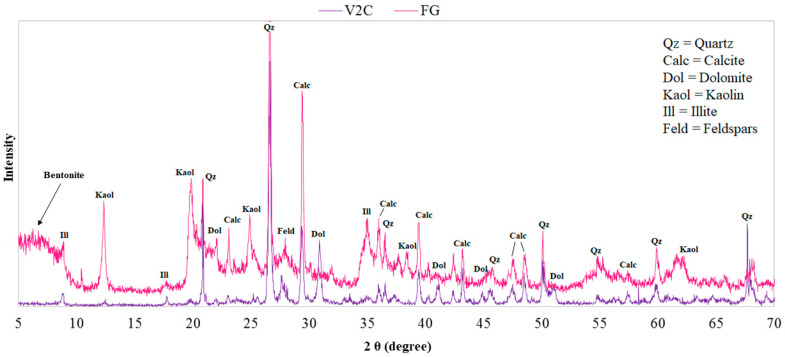
Comparison of the XRD patterns of natural SPA mud (“Grand Hotel Terme”) and “optimized” mud.

The “optimized” mud was formulated as a possible product for domestic use, also in view of maintaining a low concentration in heavy metals; therefore, the relative diffractogram was compared with that of a natural SPA mud, because the “optimized” product was realized using as a model mud the one from Euganean SPA basin for its known curative properties. In Figure 7, it can be seen that the “optimized” mud lacks minerals, such as chlorite, compared to natural SPA mud, but presents kaolinite, used in the formulation phase for its anti-caking function. In the formulation of the “optimized” mud, a small amount of bentonite has also been included, as can be seen by its small peaks between 5 and 6 2θ values [37,38].

### 3.5. List of Physical, Chemical, and Microbiological Analysis

In order to better understand the analysis pattern followed by us, it is useful to summarize the investigations that should be carried out on SPAs matrices, to characterize them from several points of view and identify a possible characteristic fingerprint.

From a physical point of view, it is essential to characterize the mud according to the particle size of grains. As mentioned in the introduction, the sludge is applied on the external surface of the body, typically with an initial coating phase. The particle size is a quality index, because too high grain sizes could trigger irritation on the skin. Mineralogical characterization is another fundamental analysis, with the sludge being a mixture of different minerals. Understanding the mineralogical composition of a natural mud could be the starting point for formulating a cosmetic product for domestic use, which maintains the same characteristics as the SPA treatment or at least would consent the benefits achieved by the SPA treatment.

From a chemical point of view, matrices should be characterized at first by the pH, always taking into consideration the application on the external surface of the body: The skin has a good buffering capacity, but a too alkaline pH damages its barrier properties. Furthermore, because the different matrices differ from one another in the chemical composition, according to the sampling area, this can be useful to verify both the quality and authenticity.

The microbiological aspect was not considered in this first part of the study but remains an important aspect currently under investigation in our laboratories. In fact, the muds from the Euganean SPAs (Abano, Montegrotto, and Battaglia Terme) are characterized by the presence of autotrophic, mixotrophic, and heterotrophic microorganisms. It is known that SPA mud is widely used in European countries for the treatment of rheumatic diseases and its therapeutic effect is ascribed to molecules deriving from microrganism metabolism, which are released during the maturation process [39].

Understanding the nature of the biological matrix is important to associate the therapeutic effect with a precise mud. Since each SPA boasts the ability to treat aspects of even very different pathologies, the characterization of the physical-chemical composition and of the microbiome would be a very useful cross-method for the study of the relationship between the composition and effectiveness of the mud itself. For this reason, characterizing the matrix also from a microbiological point of view is fundamental to understanding its possible therapeutic effects.

## 4. Conclusions

This study allowed us to deepen the knowledge about the pH, granulometry, and chemical and mineralogical composition of the SPA mud collected in the B.I.O.C.E. zone, chosen as a target for its known curative properties [3].

The peloids are characterized by a neutral/alkaline pH and are divided into two categories from a granulometric point of view: Silty-clayey and clay-silty. In particular, samples with a finer particle size are the semi-synthetic (V4C) and the optimized (FG) ones. In addition, we confirmed previously in an investigation that the maturation time does not significantly influence the grain size of the peloids [8]. The analysis of the chemical composition allowed the observation of numerous correlations between the oxides present in the samples, confirming the expected results and quantifying the presence of heavy metals, which resulted in concentrations much higher than the set reference values. In clayey materials, these elements are inevitably present, so future investigations will be directed towards two main aspects, including: (i) The mobility of the element within and from the crystalline structure of the mud, and (ii) the effective permeation through the skin, mimicking the conditions of the temperature, pH, and application time typical of a classic mud therapy session. Indeed, the presence of the element does not imply its release from the crystalline structure of the mud. Finally, mineralogical analysis made it possible to identify and compare the composition of each sample, also according to the maturation time. As expected, the characteristics do not change in such a circumscribed area as B.I.O.C.E. and the maturation time does not involve any variation from the mineralogical point of view of the samples, confirming the composition levels normally present in these types of materials [40].

In conclusion, the aim of the study was to develop a standard protocol that would allow comparisons between natural SPA muds taken from different areas and also between natural SPA muds and commercial products, formulated in the laboratory by mixing natural clays from different sources. Thanks to the method adopted, the expected results were achieved.

A totally unexpected result from this investigation regards the fact that despite the high concentration found in heavy metals (i.e., nickel), no adverse effects are usually reported by the SPA bath [11,12,13]. Considering the distribution of subjects suffering from an intolerance or allergy to heavy metal, this occurrence appears to so far be unexplained. We may advance the hypothesis that the heavy metals are sequestered within the peloids and are neither released nor come in contact with skin because they are somehow trapped. Additionally, the semi-synthetic and optimized sludge that are commercially available have all passed the safety tests on human volunteers required before commercialization. Very interestingly, this does not occour in other cosmetic products, such are, for example, emulsions containing much lower levels of heavy metals.

The perspectives for further studies, currently underway, involve the use of the data obtained to draw composition–effectiveness relationships of a natural SPA mud with that of an optimized mud, proving the dermo-cosmetic efficiency, and thus obtaining predictive models to design safe and effective “optimized” muds. In particular, this includes an investigation of the most well-known properties that are boasted: Hydration and the treatment of skin blemishes (cellulite, skin blemishes, roughness, etc.). Additionally, as reported in other studies [34,41], some variables, such as the mud bathing temperature, C-reactive protein level, and percutaneous migration of chemical elements, should be investigated in order to better understand the therapeutic effects of SPA mud treatment. Lastly, relationships between skin microbioma and mud microbioma are also undergoing. Finally, the absence of the reported incidence of allergy reactivation is also under investigation at our laboratories in collaboration with SPA centers.

## Figures and Tables

**Table 1:** Classification of peloids [6].

Peloid		
Mature mud or muddy dispersion, having health or cosmetic properties, composed of a complex mixture of a fine-grained material of geological and/or biological origin, mineral or marine water and organic compounds deriving from a biological metabolic activity.		
Origin	Composition	Application
Natural Peloid	Organic Peloid	Medical Peloid
Mud matured in the natural sedimentary environment	It contains a higer quantity of organic fraction	Specific therapeutic properties have been recognized by the national authorities that approve medicines
Peloid Strictu Sensu	Inorganic Peloid	Cosmetic Peloid
Mud matured in tanks with mineral water	It contains a higer quantity of inorganic or mineral fraction	Specific cosmetic properties have been recognized by specialized laboratories and certified in dermocosmesis
	Mixed Peloid	

**Table 2:** Sampling, provenience, and characteristics of sludges analyzed.

Sample Type	Source	Feature	
		Sample Code	Maturation Time
Natural thermal mud	Grand Hotel Terme, Montegrotto Terme (supply well: Lispida lake)	V1C	1 month
		V2C	6 months
		V3C	3 weeks
	Hotel President, Abano Terme (supply well: Costa d’Arquà lake)	V1	5 months
		V2	6 months
		V3	7 months
		V4	8 months
		V5	18 months
Semisynthetic mud	Galzignano Terme	V4C	6 months
“Optimized” mud	Commercial	FG	/

**Table 3:** Average pH of the samples analyzed.

Sample Code	Maturation Time	pH (Average Value)
V1C	1 month	7.37
V2C	6 months	7.38
V3C	3 weeks	7.52
V1	5 months	7.56
V2	6 months	7.57
V3	7 months	7.55
V4	8 months	7.50
V5	18 months	7.55
V4C	6 months	8.89
FG	/	8.00

**Table 4:** Particle size distribution expressed as a percentage of each grain size.

Granulometric Analysis		wt% Average Values			
Sample Code	Maturation Time	Sand	Silt	Clay	Shepard’s Classification
V1C	1 month	13.64	66.71	19.65	Clayey-silt
V2C	6 months	12.11	66.34	21.47	Clayey-silt
V3C	3 weeks	12.41	67.96	19.63	Clayey-silt
V1	5 months	8.97	64.44	26.47	Clayey-silt
V2	6 months	9.94	64.89	24.91	Clayey-silt
V3	7 months	9.78	65.56	24.21	Clayey-silt
V4	8 months	8.72	66.15	25.01	Clayey-silt
V5	18 months	3.07	61.27	35.61	Clayey-silt
V4C	6 months	1.65	34.07	64.29	Silty-clay
FG	/	0.82	34.74	64.45	Silty-clay

**Table 5:** Particle size distribution of the sandy fraction.

Sand’s Granulometric Distribution		wt% Average Values				
Sample Code	Maturation Time	Very Coarse Sand	Coarse Sand	Medium Sand	Fine Sand	Very Fine Sand
V1C	1 month	0.15	0.57	1.13	2.71	9.09
V2C	6 months	0.00	0.37	1.00	2.62	8.11
V3C	3 weeks	0.29	0.65	1.11	2.53	7.83
V1	5 months	0.10	0.38	0.54	1.69	6.26
V2	6 months	0.11	0.42	0.55	1.88	6.97
V3	7 months	0.09	0.44	0.59	1.79	6.86
V4	8 months	0.04	0.39	0.48	1.79	6.02
V5	18 months	0.05	0.18	0.25	0.43	2.16
V4C	6 months	0.00	0.00	0.00	0.00	1.65
FG	/	0.00	0.00	0.00	0.07	0.75
Subtotal average value		0.09	0.38	0.63	1.72	6.11

**Table 6:** Classification of the sandy component according to Udden–Wentworth [22].

Sand’s Type Classification	Particles Diameter	
Definition	[mm]	[Φ]
Very coarse sand	0	1
Coarse sand	1	0.5
Medium sand	2	0.25
Fine sand	3	0.125
Very fine sand	4	0.063

**Table 7:** Percentage values for the main elements and trace elements present in the sludge samples.

Oxides Characterization	Average Value (%)											
Sample Code	SiO_2_	TiO_2_	Al_2_O_3_	Fe_2_O_3_	MnO	MgO	CaO	Na_2_O	K_2_O	P_2_O_5_	Na_2_O + K_2_O	LOI
V1C	51.16	0.49	8.82	3.02	0.15	6.99	25.62	1.30	2.29	0.17	3.59	17.77
V2C	50.79	0.48	8.85	3.13	0.16	7.07	25.71	1.31	2.33	0.17	3.63	17.40
V3C	51.07	0.49	8.89	3.11	0.16	6.91	25.59	1.30	2.33	0.17	3.63	18.01
V1	51.70	0.50	9.20	3.47	0.19	6.77	24.63	1.09	2.27	5.37	3.34	17.42
V2	51.55	0.50	9.05	3.40	0.19	6.66	25.09	1.12	2.28	4.18	3.40	18.70
V3	51.25	0.49	8.96	3.52	0.19	6.70	25.33	1.12	2.27	5.72	3.39	19.17
V4	51.59	0.50	9.19	3.44	0.19	6.65	24.88	1.13	2.27	4.12	3.40	18.54
V5	50.90	0.51	9.27	3.79	0.23	6.62	25.15	1.07	2.34	3.66	3.41	19.68
V4C	58.62	0.80	19.90	6.56	0.16	3.37	7.23	1.80	2.19	0.09	3.27	6.70
FG	58.40	0.82	20.27	6.85	0.16	3.16	6.61	1.50	2.15	0.11	3.64	4.85

**Table 8:** Average values of the elements present in the mud samples.

Elements Characterization	Average Value (ppm)																				
Sample Code	Ba	Ce	Co	Cr	Cu	Ga	Hf	La	Nb	Nd	Ni	Pb	Rb	S	Sc	Sr	Th	V	Y	Zn	Zr
V1C	234.90	31.70	12.90	36.15	23.40	7.78	0.13	34.28	4.50	19.50	18.47	22.10	41.98	11,133.52	11.77	337.82	2.38	46.27	8.55	53.33	64.82
V2C	229.35	32.80	12.78	38.95	23.43	8.32	0.12	35.02	4.48	10.93	18.95	21.48	31.37	12,650.97	12.17	419.10	2.33	47.35	6.98	55.95	59.37
V3C	230.48	32.50	13.27	37.40	25.28	8.22	0.23	35.87	5.05	10.23	18.30	26.73	32.35	12,445.08	11.48	331.88	2.03	46.72	7.73	55.90	63.67
V1	228.10	30.23	14.70	44.53	24.20	7.87	0.20	18.50	4.10	11.70	21.57	19.47	29.13	13,649.30	12.00	269.00	4.27	53.67	6.37	73.47	47.00
V2	228.50	28.30	13.50	40.33	22.67	7.13	0.23	18.37	4.30	9.83	19.97	18.30	28.77	12,547.10	11.73	278.63	4.47	51.93	6.63	75.60	49.63
V3	224.37	28.57	14.63	39.53	25.67	8.37	0.20	21.07	4.37	9.83	32.43	20.30	33.47	12,627.87	12.47	318.80	4.23	50.30	7.40	87.90	56.50
V4	232.37	25.83	13.60	41.27	23.53	7.77	0.27	17.10	4.10	10.80	20.97	21.77	32.13	12,739.73	12.47	299.10	4.17	51.03	7.17	76.67	54.00
V5	227.00	28.27	17.37	42.37	26.90	7.83	0.23	17.53	4.03	9.83	24.27	18.00	30.73	12,573.70	12.37	260.10	4.33	58.00	5.83	120.23	39.80
V4C	445.35	60.77	23.75	106.48	54.65	23.60	4.93	58.20	12.02	23.85	55.33	24.77	73.28	5813.60	17.08	327.85	9.60	131.10	11.92	106.22	85.45
FG	350.10	64.05	24.50	122.85	55.35	23.35	5.05	38.00	11.55	26.30	57.40	26.65	70.45	9243.55	17.95	298.70	13.45	132.40	10.70	111.35	81.95
